# RNA sequencing reveals dynamic expression of genes related to innate immune responses in canine small intestinal epithelial cells induced by *Echinococcus granulosus* protoscoleces

**DOI:** 10.3389/fvets.2024.1503995

**Published:** 2024-11-29

**Authors:** Zhengrong Wang, Na Pu, Wenqing Zhao, Xuke Chen, Yanyan Zhang, Yan Sun, Xinwen Bo

**Affiliations:** ^1^State Key Laboratory of Sheep Genetic Improvement and Healthy Production, Xinjiang Academy of Agricultural and Reclamation Science, Shihezi, China; ^2^Institute of Animal Husbandry and Veterinary Medicine, Xinjiang Academy of Agricultural and Reclamation Science, Shihezi, China; ^3^College of Animal Science and Technology, Shihezi University, Shihezi, China; ^4^College of Animal Science and Technology, Tarim University, Alar, China

**Keywords:** *Echinococcus granulosus*, definitive host, innate immunity, pattern recognition receptors, RNA sequencing

## Abstract

**Background:**

Dogs are definitive hosts of *Echinococcus granulosus*, with the small intestine being the only site of parasitic infections. However, the immunomodulatory processes that occur during interactions between *E. granulosus* and its definitive host remain unclear. Therefore, this study aimed to evaluate gene transcription patterns in canine small intestinal epithelial cells (CIECs) following stimulation by *E. granulosus* protoscoleces (PSCs). Particularly, this study investigated the roles of pattern recognition receptors (PRRs), involved in recognizing pathogen-associated molecular patterns (PAMPs) and mediating the host innate immune response to the tapeworm *E. granulosus*.

**Methods:**

RNA sequencing (RNA-seq) was used to examine gene transcription patterns in CIECs following stimulation with PSCs for 12 and 24 h. The potential roles of differentially expressed (DE) genes were inferred through Gene Ontology (GO) enrichment and Kyoto Encyclopedia of Genes and Genomes (KEGG) analyses.

**Results:**

RNA-seq analysis identified 78,206,492–90,548,214 clean reads in 12 RNA samples. This included six samples stimulated with PSCs for 12 h (PSC1_12h–PSC3_12h) and 24 h (PSC1_24h–PSC3_24h) and six corresponding control samples (PBS1_12h–PBS3_12h and PBS1_24h–PBS3_24h). In the PSC_12h vs. PBS_12h and PSC_24h vs. PBS_24h groups, 3,520 (2,359 upregulated and 1,161 downregulated) and 3,287 (1765 upregulated and 1,522 downregulated) DEgenes were identified, respectively. The expression of 45 PRRs genes was upregulated in the PSC_12h and PSC_24h groups compared to those in the control groups, including 4 Toll-like receptors (TLRs), 4C-type lectin receptors (CLRs), 3 NOD-like receptors (NLRs), 17 G protein-coupled receptors (GPCRs), 4 scavenger receptors (SRs), and 13 leucine-rich repeat-containing proteins (LRRCs). GO enrichment and KEGG analyses revealed that these DEgenes were mainly involved in the regulation of host immune response processes and molecules. These included antigen processing and presentation, Th17, PI3K-Akt, Th1, and Th2 cell differentiation, neutrophil extracellular trap formation, NOD- and Toll-like receptors, TNF, intestinal immune network for IgA production and IL-17 signaling pathway. Furthermore, the identified DEgenes were involved in the regulation of signaling molecules and interaction (e.g., cell adhesion molecules and ECM-receptor interaction).

**Conclusion:**

These preliminary findings provide novel perspectives on the host innate immune response to *E. granulosus* PSC stimulation, with a focus on the involvement of *E. granulosus*-specific PRRs in host defense mechanisms against infection.

## 1 Introduction

The zoonotic and highly pathogenic tapeworm *Echinococcus granulosus* is a parasitic helminth belonging to the family Taeniidae (Platyhelminthes, Cestoda, Cyclophyllidea) (1). This tapeworm is the causative agent of cystic echinococcosis (CE), which affect at least 50 million people worldwide. CE occurs mainly in South America, Asia, Western and Central Europe, Australia, and North Africa (2). In China, CE is primarily prevalent in Xinjiang, Qinghai, Xizang, and Ningxia (3, 4). There are two types of hosts in the life cycle of *E. granulosus*: definitive and intermediate (5, 6). Carnivores such as dogs and wolves are definitive hosts. *E. granulosus* protoscoleces (PSCs) mature into adult worms within the small intestine of a definitive host following ingestion of the internal organs of an infected intermediate host (5, 6). Mammalian species such as sheep and goats are intermediate hosts that are infected by ingesting eggs released from the feces of carnivores. Similarly, humans become intermediate hosts when they ingest *E. granulosus* eggs. During the development of hydatid cysts, oncosphere larvae hatch from eggs and reside mostly in the liver and lungs of intermediate hosts. Hydatid cysts contain fluid and are enclosed by walls. PSCs arise from the innermost germinal layer of the cystic wall (5).

Canines are susceptible to infection by a variety of pathogens, including viruses, such as canine parvovirus (7), and bacteria, such as *Escherichia* species (8). Canines also act as the definitive hosts for *E. granulosus*, with infected dogs often displaying minimal clinical symptoms. Given their close interactions with humans, dogs represent a potential source for the transmission of the disease (9). The intermediate host vaccine Eg95 has been successfully developed, demonstrating a protective efficiency of up to 98%. However, the recombinant Eg95 protein does not confer protection to hosts already infected with *E. granulosus* and harboring cysts (10). Notably, the population of definitive hosts, primarily dogs, is significantly smaller compared to that of intermediate hosts, such as cattle and sheep. Therefore, vaccinating the definitive hosts would result in a substantial reduction in the overall cost of immunization. Unfortunately, there is no commercial definitive host vaccine yet. Therefore, the development of a dog vaccine against *E. granulosus* is urgently needed. Numerous studies have been conducted on vaccine candidate antigens for definitive hosts against *E. granulosus* infection. Antigens such as Myophilin (11), Tropomyosin (12), the EgM family (13), EgHCDH (14), TSP11 (15) and ANX (16) have demonstrated partial immunity and protective effects in definitive hosts. These findings collectively provide a theoretical foundation for the future development of effective vaccines against *E. granulosus*.

The limited advancements in the development of efficacious vaccines for definitive hosts can be attributed to an insufficient understanding of the immunology of *E. granulosus* infection and inadequate comprehension of the immune processes related to the elimination of intestinal parasites in dogs. Furthermore, the challenges associated with conducting immunological studies on dogs are exacerbated by the scarcity of data and tools. Moreover, these challenges are exacerbated by the absence of experimental models that can replicate the progression from the larval stage to adult worms within the intestines of definitive hosts (17). Therefore, the molecular events associated with infection in definitive hosts and immune response between *E. granulosus* and its definitive hosts warrant further elucidation. This could enable the development of an efficacious vaccine against this parasite. Transcriptomic analyses are used to identify potential diagnostic or therapeutic targets for the treatment of tumors and infectious diseases by elucidating genes of interest or biological events under specific conditions or disease states (18–20). Therefore, the present study investigated the transcriptional expression profiles and immune regulatory mechanisms of small intestinal epithelial cells in dogs following PSC stimulation. Our findings provide a theoretical basis for the development of a vaccine targeting definitive hosts.

## 2 Materials and methods

### 2.1 Ethics approval and consent to participate

This study was reviewed and approved by the Care and Use of Laboratory Animals of the Xinjiang Academy of Agricultural and Reclamation Sciences (Shihezi, China) (XAARS; Approval no. 2019–012, April 9, 2019). All animals were handled in strict accordance with the animal protection laws of the People’s Republic of China (a draft animal protection law was released on September 18, 2009) and the National Standards for Laboratory Animals in China (executed on January 5, 2002).

### 2.2 Cells

The canine small intestinal epithelial cells (CIECs) were isolated, characterized, and preserved in our laboratory (21). The small intestine of a newly born fetal dog was cleaned 3–5 times with phosphate-buffered saline (PBS) containing 1% penicillin–streptomycin under aseptic conditions. The small intestine was then cut into pieces smaller than 1 mm^3^, transferred to a 50 mL centrifuge tube, washed with PBS, and subjected to repeated pipetting and centrifugation at 100 × *g* for 7 min. The tissue pieces were then cultured using the tissue block adherence method in DMEMF12 complete medium at 37°C in a 5% CO_2_ incubator. Purification by trypsin differential digestion was performed as described previously (21). Wells exhibiting robust cell growth were selected, and upon reaching 90% confluence, the culture medium was carefully aspirated. The cells were subsequently washed three times with phosphate-buffered saline (PBS). A 0.25% trypsin solution was then applied to facilitate cell digestion. Observation under an inverted microscope revealed that the fibroblasts exhibited shrinkage and increased refractivity, at which point the trypsin was removed. The digestion process was halted by the addition of DMEM/F2 medium supplemented with 5% fetal bovine serum. Following several pipetting cycles, the majority of fibroblasts were successfully detached, whereas the canine small intestinal epithelial cells remained adherent.The DMEM/F12 medium supplemented with 5% fetal bovine serum was replenished in the culture. After 48 h, if fibroblasts remained present, the aforementioned purification method was reapplied until the majority of cells within the field of view were epithelial cells. A fluorescence immunoassay was used to detect the molecular markers of epithelial cells to ascertain the identity of the isolated cells as small IECs (21, 22). First harvest cells in the logarithmic growth phase and inoculate 1 × 10^4 cells per well into 24-well cell culture plates. Once the cells have adhered to the plates, wash them 2–3 times with phosphate-buffered saline (PBS). Fix the cells with 4% paraformaldehyde for 15 min, followed by three washes with PBS. Subsequently, permeabilize the cells using 0.5% Triton X-100 at room temperature for 1 min, and rinse them twice with PBS. Apply a 4% bovine serum albumin (BSA) blocking solution and incubate at room temperature for 30 min. Remove the blocking solution and introduce the mouse anti-keratin 18 monoclonal antibody, diluted at 1:100. Following three washes with PBS, introduce the goat anti-mouse FITC-conjugated secondary antibody, diluted at a ratio of 1:200, and incubate at 37°C in the dark for 1 h. Subsequently, perform three additional washes with PBS, add DAPI, and incubate in the dark for 3 min. Conclude with a single PBS wash and proceed to immediate observation under a fluorescence microscope. The canine small intestinal epithelial cells were stained in green fluorescence. Upon confirmation of these results, the cells were cultured for subsequent experiments.

### 2.3 Preparation of PSCs

Hydatid cysts were collected from the livers of naturally infected sheep at an abattoir in Shihezi, Xinjiang Province, China. PSCs were aspirated aseptically from the hydatid cysts, centrifuged at 1000 × *g* for 15 min, and digested with pepsin. The G1 genotype of PSCs was identified as previously described (23). The viability of the PSC was determined through staining with 0.1% methylene blue; dead PSCs were stained blue. The PSCs were subsequently cultured in RPMI 1640 medium (Gibco, Auckland, New Zealand) containing 25% (v/v) fetal bovine serum (Gibco), 100 U/mL penicillin, and 100 μg/mL streptomycin in a culture flask at 37°C in the presence of 5% CO_2_ (24).

### 2.4 *In vitro* stimulation of canine IECs

5.0 × 10^6^ canine IECs were stimulated for 12 h or 24 h with 2000 PSCs (experimental groups: PSC1_12h to PSC3_12h) or 24 h (experimental groups: PSC1_24h to PSC3_24h), The Canine IECs without stimulation of PSC were collected as control groups at 12 h (controls groups: PBS1_12h to PBS3_12h) or 24 h (control groups: PBS1_24h to PBS3_24h). All experiments were conducted in triplicate to ensure biological replicates. Cells in all experimental and control groups were collected into TRIzol and stored at −80°C until RNA extraction.

### 2.5 RNA extraction and qualification analysis

Total RNA was extracted from each sample using an RNA Isolation Kit (Invitrogen, Carlsbad, CA, USA) according to the manufacturer’s instructions. Cells were collected via trypsin digestion and treated with Trizol reagent and chloroform. After centrifuging at 12,000 g and 4°C for 15 min, the upper aqueous phase was transferred to a new tube. Isopropanol was added, and the mixture was centrifuged again under the same conditions for 10 min. The supernatant was discarded, and the precipitate was washed with 75% ethanol. The RNA precipitate was dried at room temperature, then dissolved in DEPC water. The solution was aliquoted into clean tubes and stored at −70°C. The RNA was monitored for degradation and contamination on a 1% agarose gel. The purity of the extracted RNA was determined using a NanoPhotometer spectrophotometer (IMPLEN, CA, USA). The RNA concentration and integrity were measured using a Qubit RNA Assay Kit in a Quit 2.0 Fluorometer (Life Technologies, Carlsbad, CA, USA) and an RNA Nano 6,000 Assay Kit of the Bioanalyzer 2,100 system (Agilent Technologies, CA, USA). RNA samples with a 28S:18S ratio ≥ 0.7 and RNA integrity number ≥ 7 were used for further analysis.

### 2.6 Library construction, quality control, and sequencing

Sequencing libraries were generated using the NEBNext Ultra Directional RNA Library Prep Kit (NEB E7420; Illumina) according to the manufacturer’s instructions. Index codes were added to attribute sequences to each sample. Next, rRNA was removed from the total RNA sample (animal- TruSeq Stranded Total RNA Library Prep Gold/Illumina/20020599). Fragmentation was then carried out using divalent cations under elevated temperatures in the First Strand Synthesis Reaction Buffer (5X). First-strand complementary DNA (cDNA) was synthesized using random hexamer primers and MMuLV Reverse Transcriptase (RNaseH). Second-strand cDNA synthesis was subsequently performed using DNA polymerase I and RNase H. The remaining overhangs were converted into blunt ends via exonuclease/polymerase activity. After adenylation of 3′ ends of the DNA fragments, NEBNext Adaptors with a hairpin loop structure were used to ligate the fragments to prepare for hybridization. The library fragments were purified using the AMPure XP system (Beverly, USA) to select DNA fragments 370–420 bp in length. Then, 3 μL USER Enzyme (NEB, USA) was used with size-selected, adaptor-ligated cDNA at 37°C for 15 min, followed by 5 min at 95°C before PCR. PCR was performed using the Phusion High-Fidelity DNA polymerase, Universal PCR primers, and Index (X) Primer. Finally, PCR products were purified (AMPureXP system), library quality was assessed on an Agilent 5,400 system (Agilent Technologies), and quantification was performed using QPCR (1.5 nM). Novogene Bioinformatics Technology Co., Ltd. (Beijing, China) pooled and sequenced the qualified libraries on Illumina platforms using the PE150 strategy according to the effective library concentration and amount of data required.

### 2.7 Bioinformatic analysis pipeline

#### 2.7.1 Quality control

The raw reads obtained by RNA-seq were processed by using SortMeRNA software to remove ribosomal RNA (rRNA) sequences, and reads with low-quality were filtered by using Trimmomatic software. Clean reads were obtained by trimming reads containing adapters or poly-N, or those with low quality from raw data. Simultaneously, Fastp software was then used to assess the quality of filtered reads through setting a number of important parameters, such as the length distribution, Q20, Q30, and GC contents. All downstream analyses were performed using clean high-quality data.

#### 2.7.2 Read mapping to the reference genome

The reference genome and gene model annotation files were downloaded directly from the reference genome database.^1^ The index of the reference genome was built using Hisat2v2.0.5 and clean paired-end reads were aligned with the reference genome using Hisat2 v2.0.5. We selected Hisat2 as the mapping tool because it can generate a database of splice junctions based on the gene model annotation file and thus, provides a better mapping result than other non-splice mapping tools.

#### 2.7.3 Quantification of gene expression levels

(StringTie-1.3.3b) was used to count the number of reads mapped onto each gene. The number of fragments per kilobase of transcript sequence per million reads mapped (FPKM) was then calculated, and the expected FPKM was sequenced. FPKM is a simple and commonly used expression normalization method. It normalizes for both sequencing depth and genome size.

#### 2.7.4 Differential expression analysis

Before differential gene expression analysis, read counts were adjusted using the edgeR program package through one scaling normalized factor for each sequenced library. Differential expression analyses were performed using the EdgeR R package (version 3.22.5). *p*-values were adjusted using the Benjamini-Hochberg method. Significantly differentially expressed genes were screened based on the following criteria: corrected *p* < 0.05 and |log2FoldChange| > 1.

#### 2.7.5 Gene ontology (GO) and Kyoto encyclopedia of genes and genomes (KEGG) enrichment analyses of differentially expressed genes

GO enrichment analysis of differentially expressed genes was performed using the clusterProfiler R package, in which gene length bias was corrected. GO terms with corrected *p* < 0.05 were considered significantly enriched by differentially expressed genes. KEGG is a database resource for elucidating the high-level functions and utilities of biological systems such as cells, organisms, and ecosystems from molecular-level information, especially large-scale molecular datasets generated through genome sequencing and other high-throughput experimental technologies.^2^ The cluster Profiler R package was used to evaluate the statistical enrichment of differentially expressed genes in KEGG pathways.

#### 2.7.6 Quantitative real-time PCR analysis

Twelve CIEC samples with (experimental group) or without (control group) *E. granulosus* PSC stimulation for 12 or 24 h were collected to verify the accuracy of the RNA sequencing (RNA-seq) data using quantitative real-time PCR (qRT-PCR). Total RNA was extracted using TRIzol reagent and reverse-transcribed to cDNA using the EVO M-MLV RT Kit with gDNA Clean for qPCR II (Accurate Biotechnology Co., Ltd., Hunan, China) according to the manufacturer’s instructions. Next, qRT-PCR was performed using 2 × Universal SYBR Green Fast qPCR Mix (ABclonal, Wuhan, China). The primer sequences designed using DNAMAN 7.0 software (Lynnon Biosoft, Quebec City, Canada) are listed in Supplementary Table S1. The glyceraldehyde-3-phosphate dehydrogenase gene (GAPDH) was used as an internal reaction control, and triplicate assays were performed for each gene (21). The relative expression of each gene was calculated by using the 2 ^−ΔΔCt^ method (25).

### 2.8 Statistical analyses

Relative expression levels of selected genes in the experimental and control groups were analyzed using GraphPad PRISM (v8.0.1; GraphPad Software Inc., San Diego, CA, USA). *p* < 0.05 was considered statistically significant and determined using a two-tailed *t*-test and a parametric test.

## 3 Results

### 3.1 Transcriptomic sequencing data analysis

In total, 991,124,718 raw reads were generated from 12 samples through RNA-seq. After eliminating the low-quality reads, 972,465,930 clean reads were obtained. The clean reads exhibited quality scores (Q20 and Q30) of 97.35–98.16% and 92.75–94.10%, respectively, and average GC content of 46.44% (Supplementary Table S2). The percentage of total mapped clean reads for each sample was 94.33–95.53%, and the percentage of unique mapped clean reads for each sample was 89.39–91.15% (Supplementary Table S2). The original data were deposited in the NCBI repository under accession number PRJNA1122995.

### 3.2 Differentially expressed genes profiles

**Figure 1:**
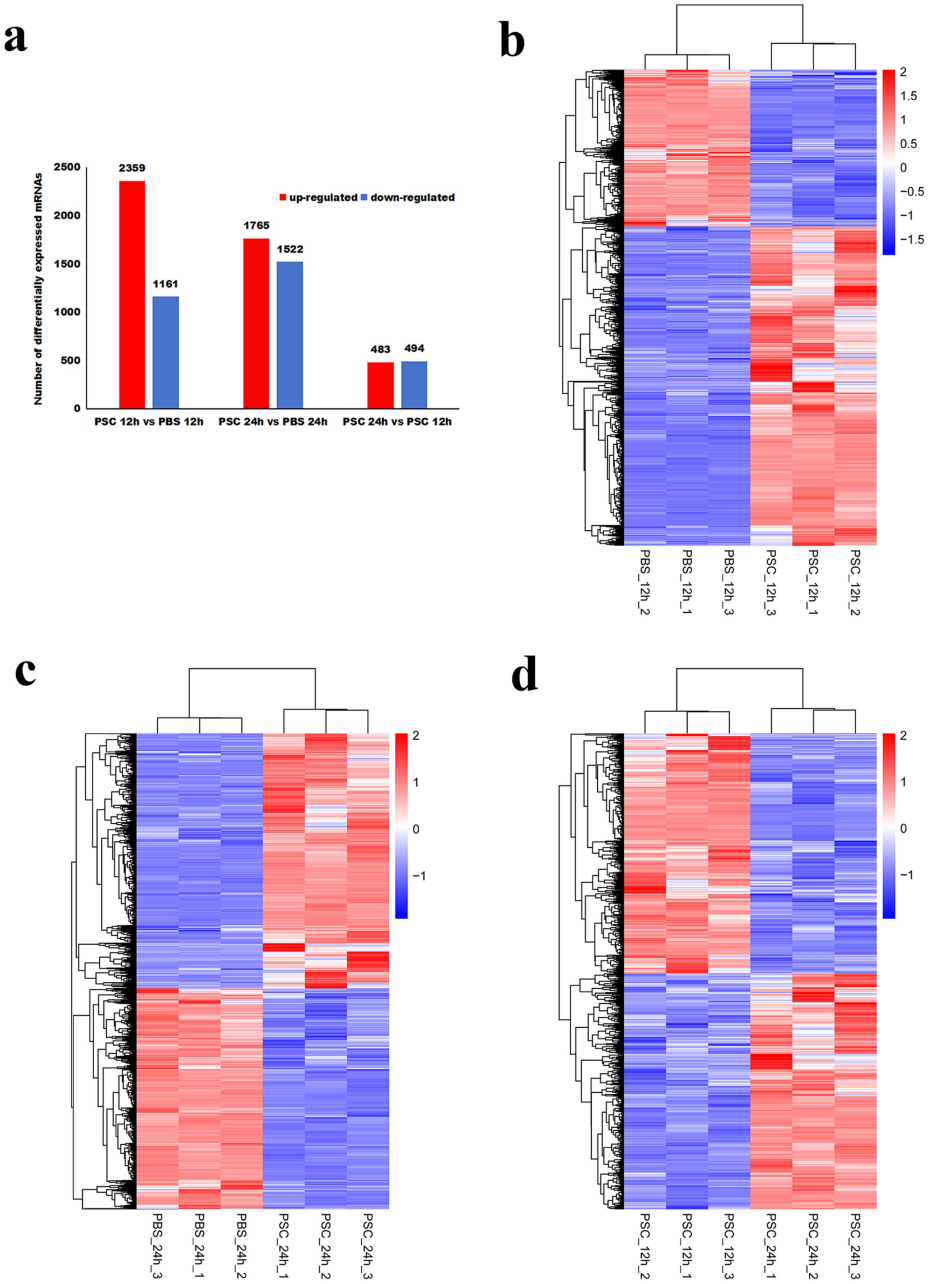
Differentially expressed genes (DEgenes) and hierarchical clustering heatmaps of the DEgenes in canine small intestinal epithelial cells (CIECs) following *E. granulosus* protoscoleces (PSC) stimulation. **(a)** The number of DEgenes. **(b–d)** Hierarchical clustering heatmaps of the DEgenes in the PSC 12 h vs. PBS 12 h (b), PSC 24 h vs. PBS 24 h **(c)**, and PSC 24 h vs. PSC 12 h **(d)** groups. *p*-values were adjusted using the Benjamini and Hochberg method, with *p* < 0.05 and Log2|Fold Change| > 1 considered statistically significant.

To evaluate the profiles of differentially expressed genes, the 12 samples were categorized into three groups: PSC vs. PBS 12 h, PSC vs. PBS 24 h and PSC 12 h vs. PSC 24 h. In total, 3,520 differentially expressed genes (2,359 upregulated and 1,161 downregulated) were identified in the PSC 12 h vs. PBS 12 h group (Figure 1; Supplementary Table S3). A total of 3,287 differentially expressed genes (1,765 upregulated and 1,522 downregulated) were identified in the PSC 24 h vs. PBS 24 h group (Figure 1; Supplementary Table S4). Similarly, 977 differentially expressed genes (483 upregulated and 494 downregulated) were identified in the PSC 24 h vs. PSC 12 h (Figure 1; Supplementary Table S5).

**Figure 2:**
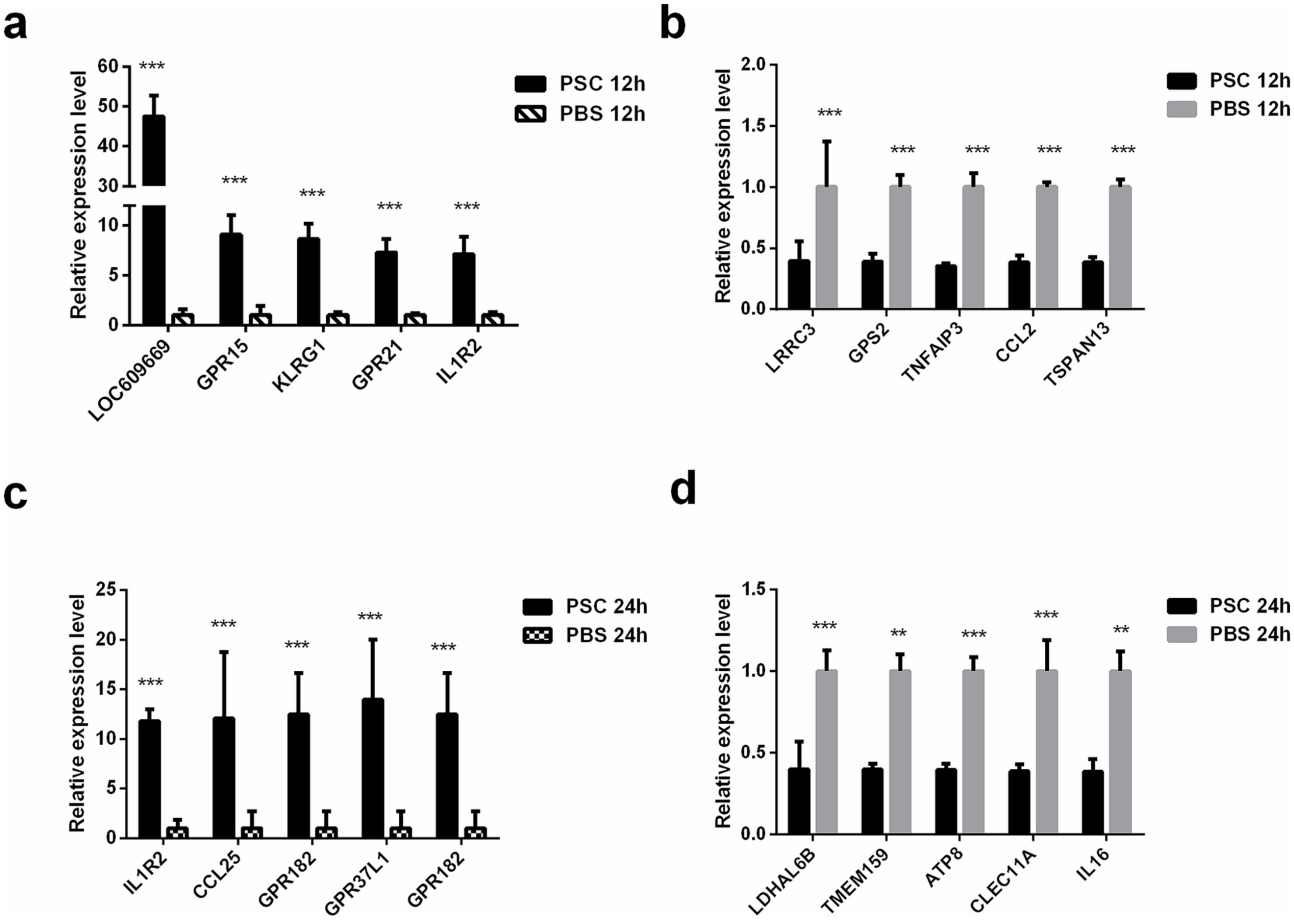
Validation of the DEgenes using quantitative real-time PCR (qRT-PCR). The validation results for up regulated genes in the PSC 12 h vs. PBS 12 h **(a)**, down regulated genes in the PSC 12 h vs. PBS 12 h **(b)**, up regulated genes in the PSC 24 h vs. PBS 24 h **(c)** and down regulated genes in the PSC 24 h vs. PBS 24 h **(d)** groups. *p < 0.05, **p < 0.01, ***p < 0.001.

Ten (five upregulated and five downregulated) genes were randomly selected for qRT-PCR analysis to verify the reliability of the RNA-seq data both in the PSC 12 h vs. PBS 12 h and PSC 24 h vs. PBS 24 h groups, respectively. The mRNA transcription levels of *LOC609669*, *GPR15*, *KLRG1*, *GPR21*, *IL1R2*, *LRRC3*, *GPS2*, *TNFAIP3*, *CCL2*, and *TSPAN13* in the PSC 12 h vs. PBS 12 h groups and *IL1R2*, *CCL25*, *GPR182*, *GPR37L1*, *GPR182*, *LDHAL6B*, *TMEM159*, *ATP8*, *CLEC11A*, and *IL16* in the PSC 24 h vs. PBS 24 h groups were consistent with the transcriptomic data, indicating high reproducibility and correctness of the transcriptomic data via RNA-seq (Figure 2).

### 3.3 GO enrichment analysis of differentially expressed genes

**Figure 3:**
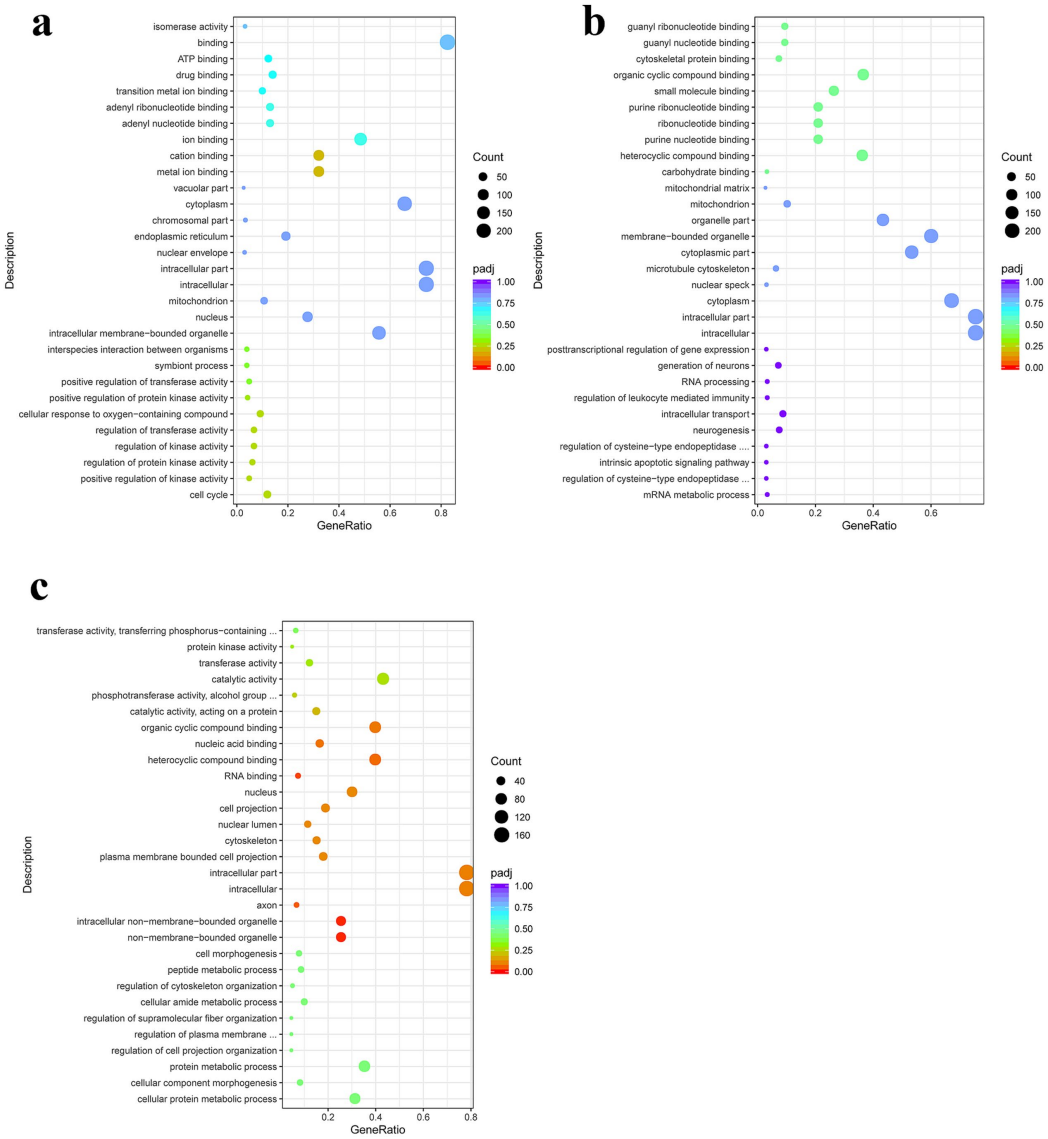
Gene Ontology (GO) enrichment analysis of the DEgenes in CIECs following *E. granulosus* PSC stimulation. **(a–c)** The GO terms enriched with DEgenes in the PSC 12 h vs. PBS 12 h **(a)**, PSC 24 h vs. PBS 24 h **(b)**, and PSC 24 h vs. PSC 12 h **(c)** groups. *p* < 0.05 and Log2|Fold Change| > 1 were considered statistically significant.

GO enrichment analysis of the differentially expressed genes in the PSC vs. PBS at 12 h showed that differentially expressed genes in the biological category were mainly enriched in the cell cycle, positive regulation of kinase activity, regulation of protein kinase activity, and regulation of kinase activity, respectively. In the molecular function category, differentially expressed genes were enriched for metal ion, cation, ion, and adenyl nucleotide binding, respectively. In the cellular component category, differentially expressed genes were mainly enriched in intracellular membrane-bound organelles, nuclei, mitochondria, and intracellular organelles, respectively (Figure 3; Supplementary Table S6). GO enrichment analysis of the PSC 24 h vs. PBS 24 h group showed that differentially expressed genes in the biological process category were mainly enriched in metabolic processes, regulation of cysteine-type endopeptidase activity involved in apoptotic processes, intrinsic apoptotic signaling pathways, and regulation of cysteine-type endopeptidase activity. In the molecular function category, 9, 106, 61, and 61 differentially expressed genes were enriched in carbohydrates, heterocyclic compounds, purine nucleotides, and ribonucleotide binding, respectively. In the cellular component category, 234, 234, 208, and nine differentially expressed genes were mainly enriched in the intracellular, intracellular part, cytoplasm, and nuclear speck, respectively (Figure 3; Supplementary Table S7).

### 3.4 KEGG enrichment analysis of differentially expressed genes

**Figure 4:**
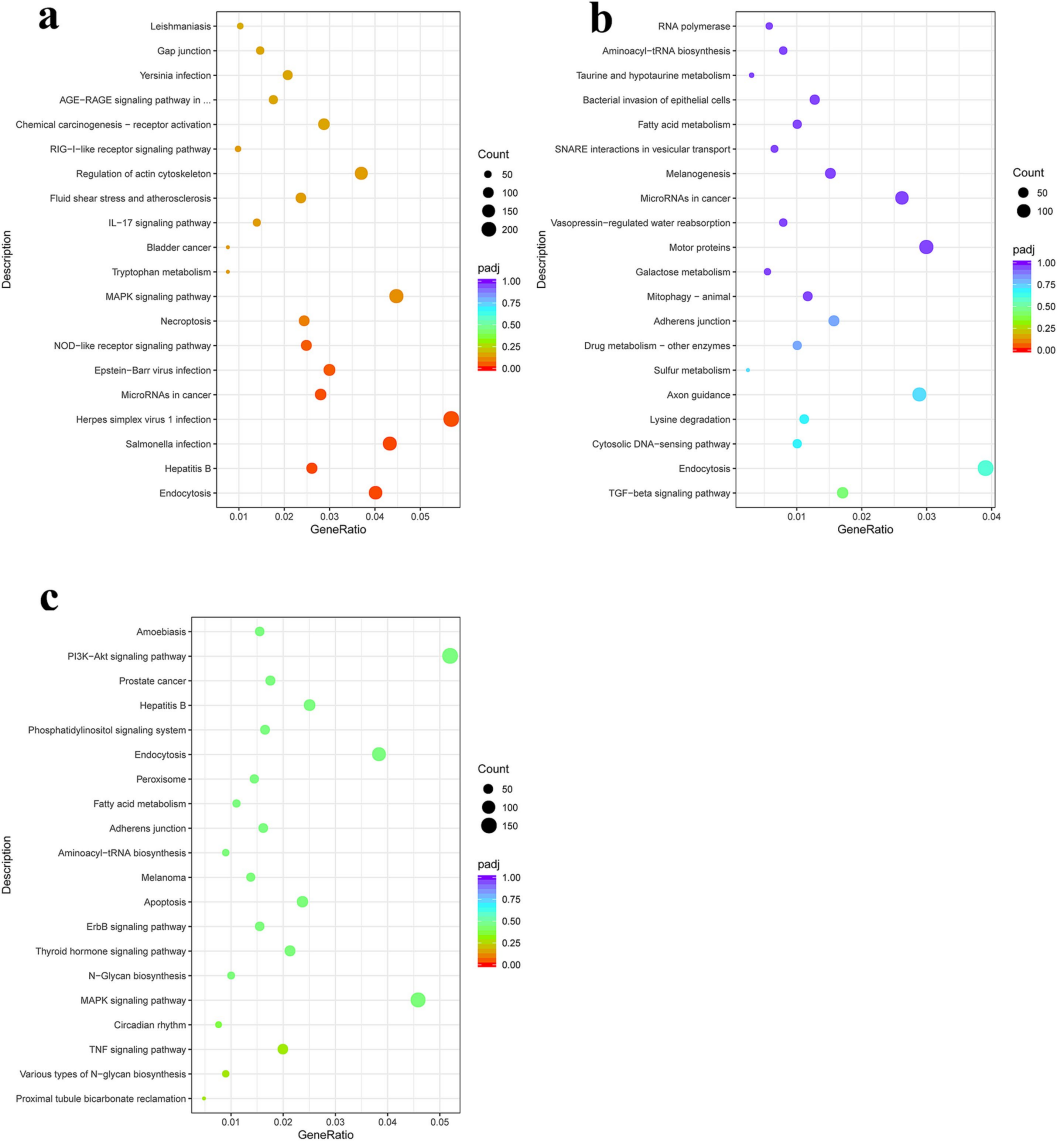
Kyoto Encyclopedia of Genes and Genomes (KEGG) pathway enrichment analysis of the DEgenes in CIECs following *E. granulosus* PSC stimulation. **(a–c)** The KEGG pathway terms enriched with DEgenes in the PSC 12 h vs. PBS 12 h **(a)**, PSC 24 h vs. PBS 24 h **(b)**, and PSC 24 h vs. PSC 12 h **(c)** groups. *p* < 0.05 and Log2|Fold Change| > 1 were considered statistically significant.

KEGG pathway enrichment analysis in the PSC vs. PBS 12 h group showed that genes with upregulated expression were mainly enriched in protein digestion and absorption, ECM-receptor interaction, herpes simplex virus 1 infection, olfactory transduction, asthma, cell adhesion molecules, antigen processing and presentation, inflammatory bowel disease, Th17 cell differentiation, the PI3K-Akt signaling pathway, as well as the Th1 and Th2 cell differentiation signaling pathways. In contrast, the genes with downregulated expression were mainly enriched in alcoholism, hepatitis B, neutrophil extracellular trap formation, as well as NOD-like receptor, RIG-I-like receptor, TNF, and Toll-like receptor signaling pathways (Figure 4; Supplementary Table S8). In the PSC 24 h vs. PBS 24 h group, KEGG pathway enrichment analysis showed that genes with upregulated expression were mainly enriched in protein processing in the endoplasmic reticulum, protein export, the IL-17 signaling pathway, antigen processing and presentation, Th17 cell differentiation, intestinal immune network for IgA production, as well as Th1 and Th2 cell differentiation signaling pathways. In contrast, the genes with downregulated expression were mainly enriched in lysine degradation, axon guidance, endocytosis, hepatitis B, as well as the Hippo, TGF-beta, NOD-like receptor, and PI3K-Akt signaling pathways (Figure 4; Supplementary Table S9).

### 3.5 PSCs upregulated or downregulated the expression of several germline-encoded receptors of canine IECs

**Figure 5:**
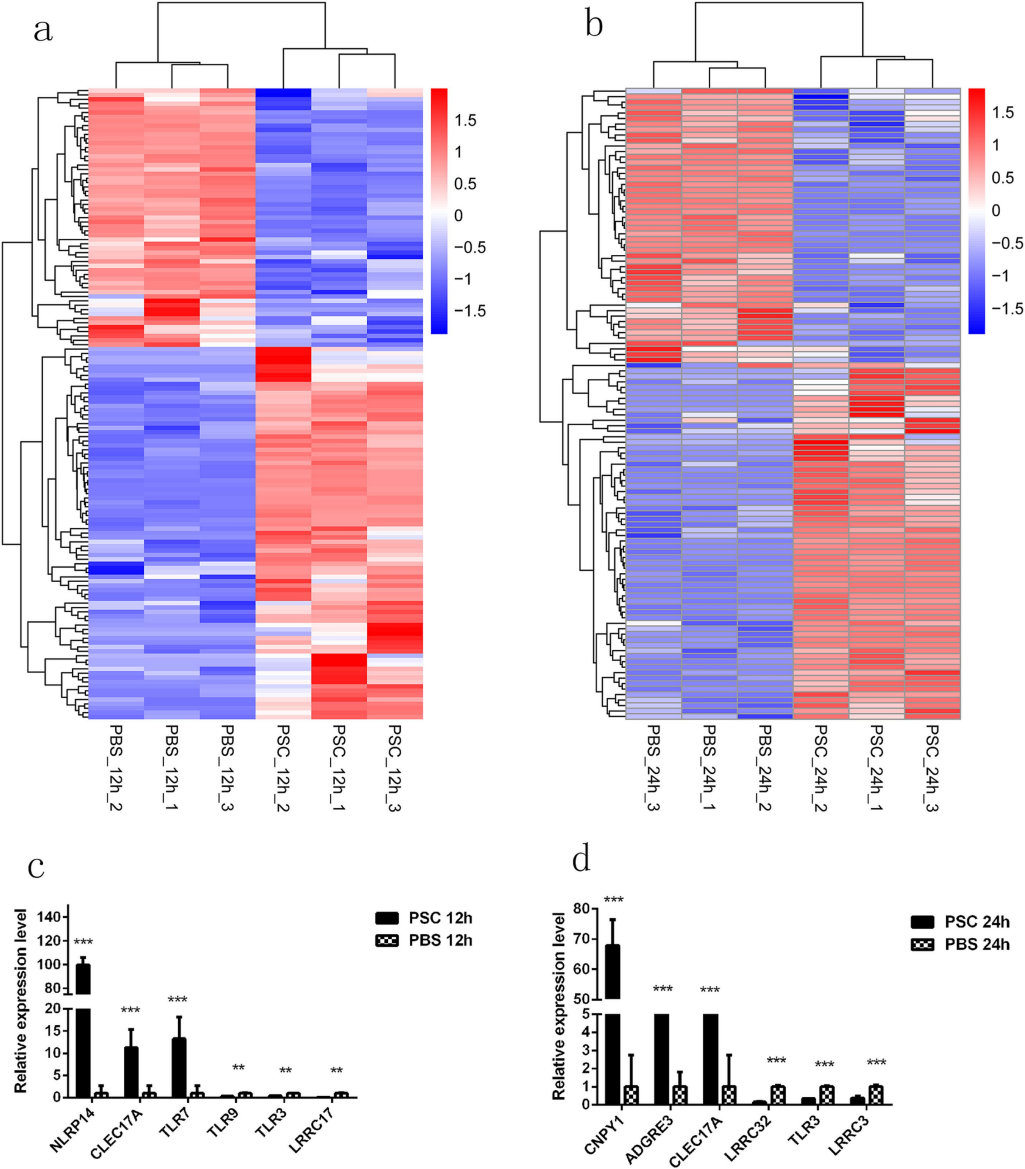
Differentially expressed germline-encoded receptors of CIECs. Hierarchical clustering heatmaps of the germline-encoded receptors in the PSC 12 h vs. PBS 12 h **(a)** and PSC 24 h vs. PBS 24 h **(b)** groups. *p* < 0.05 and Log2|Fold Change| > 1 were considered statistically significant. The qRT-PCR validation results for differentially expressed germline-encoded receptors in the PSC 12 h vs. PBS 12 h **(c)** and PSC 24 h vs. PBS 24 h **(d)** groups. *p < 0.05, **p < 0.01, ***p < 0.001.

To evaluate the potential impact of PSCs on the expression of pattern recognition receptors (PRRs) in canine IECs, transcripts associated with germline-encoded receptors were analyzed. After stimulation with PSCs, canine IECs exhibited either increased or decreased expression of various germline-encoded receptors, including Toll-like (TLRs), C-type lectin (CLRs), NOD-like (NLRs), G protein-coupled (GPCRs), and scavenger (SRs) receptors, as well as some undefined leucine-rich repeat-containing proteins (LRRCs). In total, 144 differentially expressed germline-encoded receptors were identified in the PSC 12 h vs. PBS 12 h group, including 12 TLRs (six upregulated, six downregulated), 10 CLRs (nine upregulated, one downregulated), five NLRs (three upregulated, two downregulated), 59 GPCRs (34 upregulated, 25 downregulated), 12 SRs (eight upregulated, four downregulated), and 46 LRRCs (25 upregulated, 21 downregulated) (Figure 5; Supplementary Table S10). Furthermore, 116 differentially expressed germline-encoded receptors were identified in the PSC 24 h vs. PBS 24 h group, including 16 TLRs (seven upregulated, nine downregulated), five CLRs (four upregulated, one downregulated), six NLRs (three upregulated, three downregulated), 43 GPCRs (26 upregulated, 17 downregulated), 12 SRs (eight upregulated, four downregulated), and 34 LRRCs (18 upregulated, 16 downregulated) (Figure 5; Supplementary Table S10). The mRNA transcription levels of *NLRP14*, *CLEC17A*, *TLR7*, *TLR3*, and *LRRC17* in the PSC 12 h vs. PBS 12 h groups and *CNPY1*, *ADGRE3*, *CLEC17A*, *TLR3*, and *LRRC32* in the PSC 24 h vs. PBS 24 h groups were verified with the qRT-PCR analysis.

The expression of several PRRs genes was upregulated in both the PSC 12 h vs. PBS 12 h and PSC 24 h vs. PBS 24 h groups. This included 4 TLRs (*TLR1*, *TLR6*, *TLR7*, and *TLR10*), 4 CLRs (*CLEC4A*, *CLEC17A*, *LOC611536*, and *LOC100683252*), 3 NLRs (*NLRP3*, *NLRC3* and *NLRP14*), 17 GPCRs (*ADGRF3*, *ADGRE3*, *GRK7*, *ADGRV1*, *GPRC5D*, *GPR160*, *ADGRG5*, *ADGRG7*, *GPR107*, *GPR61*, *LOC484898*, *GPR151*, *ADGRG3*, *GPR182*, *GPR135*, *GPR89A*, and *GPR15*), 4 SRs (*PRSS12*, *LOC611565*, *TMPRSS5*, and *LOXL2*), and 13 LRRCs (*LRRC6*, *LRRC23*, *LRRC36*, *LRRC4C*, *LOC106558527*, *LRRC69*, *LRRC66*, *LRRCC1*, *LRRC39*, *LRRC8B*, *LRRC9*, *LRRC53*, and *NRROS*).

### 3.6 PSCs upregulated the expression of several PRRs downstream signaling molecules in canine IECs

**Figure 6:**
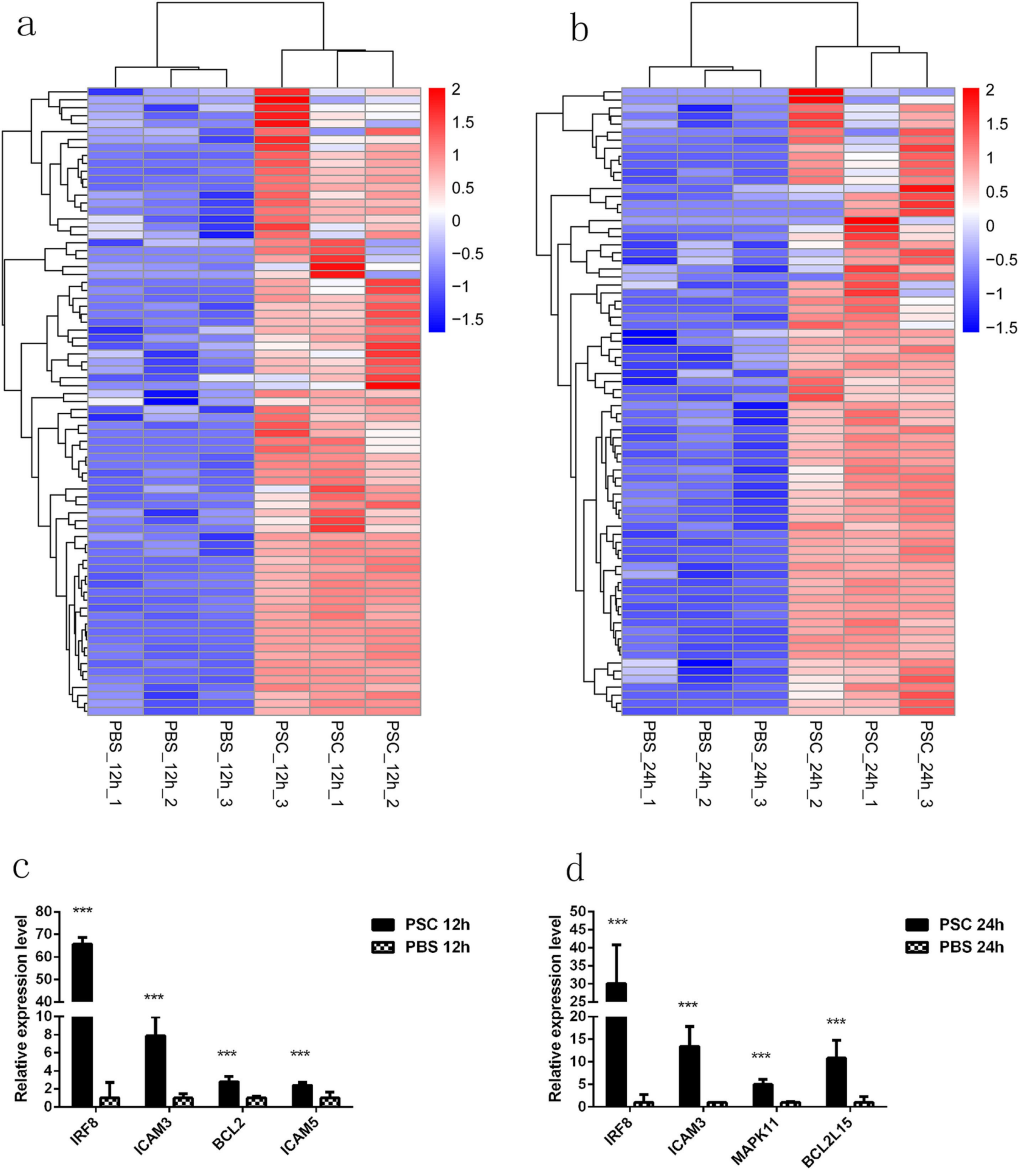
PRRs downstream signaling molecules with upregulated expression in CIECs. Hierarchical clustering heatmaps of the PRR downstream signaling molecules in the PSC 12 h vs. PBS 12 h **(a)** and PSC 24 h vs. PBS 24 h **(b)** groups. *p* < 0.05 and Log2|Fold Change| > 1 were considered statistically significant. The qRT-PCR validation results for upregulated PRRs downstream signaling molecules in the PSC 12 h vs. PBS 12 h **(c)** and PSC 24 h vs. PBS 24 h **(d)** groups. *p < 0.05, **p < 0.01, ***p < 0.001.

This study further investigated the potential effect of PSCs on downstream signaling molecules associated with PRRs. After stimulation with PSCs, the expression of essential signal transduction molecules involved in PRR signaling was upregulated in canine IECs (Figure 6; Supplementary Table S11). Furthermore, PSCs upregulated the expression of negative regulators of inflammation (*ATF6*, *BCL2*, and *BCL3*) and transcription factors involved in *IRF6*, *IRF8*, *NFATC1*, *NFATC2*, and *NFATC3* expression. Analysis of the effector molecules induced by PSCs demonstrated the upregulated expression of *IL-5*, *IL-11*, *IL-13*, *IL-16*, *IL-23A*, *IL-25*, *IL-27*, *IL-29L*, and *IL-37* in the PSC 12 h vs. PBS 12 h group. Similarly, the expression of *IL-5*, *IL-11*, *IL-13*, *IL-25*, and *IL-27* was upregulated in the PSC 24 h vs. PBS 24 h group (Figure 6; Supplementary Table S11). In addition to the differentially expressed cytokines, PSCs promoted the expression of effector molecules related to host immune responses to helminth infection. These included prostaglandins (*PTGER1*, *PTGER2*, *PTGER3*, *PTGER4*, *PTGS1*, *PTGES2*, *PTGER3*, *PTGR2*, and *PTGDR2*), MMPs (*MMP9*, *MMP13*, *MMP21*, *MMP25*, and *MMP24OS*), and pentraxins (*NPTX1*) (Figure 6; Supplementary Table S11). Furthermore, the expression of numerous genes associated with apoptosis inhibition, including *BCL2*, *BCL2L2*, *BCL2L13*, *BCL2L15*, and *BCL11B*, was upregulated in the PSC-stimulated group. Moreover, the expression of genes involved in the induction of pyroptosis, such as *NLRP3*, *NLRC3*, *NLRP14*, *GSDMB*, *GSDME*, *CASP3*, and *CASP7*, was upregulated. The mRNA transcription levels of *IRF8*, *ICAM3*, *BCL2*, and *ICAM5* in the PSC 12 h vs. PBS 12 h groups and *IRF8*, *ICAM3*, *MAPK11*, and *BCL2L15* in the PSC 24 h vs. PBS 24 h groups were verified with the qRT-PCR analysis.

## 4 Discussion

Adult *E. granulosus* worms inhabit the small intestines of dogs. Therefore, the efficacy of the local immune response in the host small intestine is important for both parasite removal and successful parasitization (26). The intestinal tract is a highly competitive ecological niche, with helminth infections negatively affecting digestive processes. Intestinal parasites have evolved mechanisms to exploit hosts and gain a competitive edge in this nutrient-rich environment (27). Moreover, attachment organs of endoparasite worms often trigger considerable inflammation (28). Inflammation is a vital host defense mechanism triggered by the presence of foreign organisms or physical injury. This process protects the host by inducing specific chemical and morphological changes in affected tissues (29, 30). Persistent parasitism of enteric helminths results in chronic infections, leading to the progression of the initial immune response toward a chronic state characterized by pathological alterations in the gut tissue (31). Epithelial cells are the predominant cellular components of the small intestine and are integral to host defense against intestinal pathogens (32). However, the role of canine small IECs in combating *E. granulosus* infections remains largely understudied. In this study, we performedmRNA transcription profiling of innate immune-related genes in canine small IECs in response to PSC stimulation.

PRRs play a crucial role in the innate immune response by recognizing pathogen-associated molecular patterns (PAMPs) and initiating intracellular signaling cascades that lead to the secretion of inflammatory cytokines and chemokines. This results in the elimination of the invading pathogens and infected cells. The main types of PRRs include TLRs, NLRs, RLRs, CLRs, GPCRs, SRs, and LRRCs (33). In the present study, PSCs regulated the gene expression of *TLR1*, *TLR2*, *TLR3*, *TLR4*, *TLR5*, *TLR6*, *TLR7*, *TLR9*, and *TLR10* in canine IECs. Specifically, the gene expression of *TLR1*, *TLR6*, *TLR7*, and *TLR10* was upregulated, whereas that of *TLR2*, *TLR3*, *TLR4*, *TLR5*, and *TLR9* was downregulated in the PSC-stimulated group compared with the PBS group. Numerous studies have investigated the correlation between *TLR1* polymorphisms and various diseases (34–36). In the present study, *TLR1* exhibited ubiquitous expression at levels markedly higher than those observed for other TLR genes.

*TLR6* is found on the cell membrane and is an essential heterodimeric partner of *TLR2*. The TLR2-TLR6 heterodimer can recognize several ligands, such as bacterial diacyl lipoproteins and heat shock proteins (37, 38). Similarly, *TLR7* plays a critical role in the innate immune response, particularly in the recognition of pathogenic patterns. It is primarily localized to the endoplasmic reticulum and endosomes, where it detects single-stranded RNA. Upon binding to its ligand, *TLR7* initiates the Myd88-dependent signaling pathway, leading to the activation of an immune response (39). The ligands and functions of *TLR10*, a transmembrane glycoprotein predominantly expressed in immune cell-rich tissues, are poorly understood. However, conflicting evidence exists regarding its proinflammatory and anti-inflammatory properties (40, 41).

Consistent with our results, a decrease in the expression of *TLR2* and *TLR4* was observed in mouse peripheral blood mononuclear cells after the exposure of PSCs to excretory-secretory and somatic antigens (42). Additionally, glycomolecules in *E. granulosus* cyst fluid can disrupt the *TLR4*-mediated activation of dendritic cells (43). However, the expression of *TLR4* was upregulated in the peripheral blood mononuclear cells (PBMCs) of water buffaloes during early infection with *Fasciola gigantica* (44). Additionally, a structural resemblance has been identified between the glycolipid AL-II of *F. hepatica* and bacterial lipid A, potentially explaining the comparable recognition of both molecules by *TLR4* and *TLR2* (45). Thus, some researchers have hypothesized that *F. gigantica*-specific PAMP is recognized by *TLR4* (44). However, the specific TLRs responsible for recognizing *E. granulosus*-specific PAMPs have not yet been identified. The differential transcription levels of TLRs following PSC stimulation suggest that TLRs with upregulated expression (*TLR1*, *TLR6*, *TLR7* and *TLR10*) may be involved in the recognition of *E. granulosus*-specific PAMPs, distinct from those involved in the recognition of *F. gigantica*-specific PAMPs.

The present study further showed that PSC stimulation can activate the regulation of other PRRs in epithelial cells of the host small intestine. NLRs are crucial microbial sensors that play a major role in the overall immune response against pathogens and aid in the successful resolution of infections (46). In this study, PSC stimulation upregulated the expression of *NLRP3*, *NLRC3* and *NLRP14* in canine IECs. The NLRP3 inflammasome is the most extensively studied inflammasome, as it can be effectively activated by diverse PAMPs, danger-associated molecular patterns, and microbial invasion (47, 48). This intracellular multiprotein complex consists of the adaptor molecule, apoptosis-associated speck-like protein containing CARD (ASC), and procaspase-1. *NLRP3* functions as a scaffold for the protein complex, and ASC facilitates the connection between *NLRP3* and the effector protein procaspase-1, leading to its activation. This activation results in the cleavage of precursor forms of *IL-1β* and *IL-18* into their active forms (49).

The inhibitory impact of schistosomal egg antigens on TLR signaling is concomitantly linked to the stimulation of the NLRP3 inflammasome, resulting in the production of IL-1β (50). Furthermore, activation of the NLRP3 inflammasome in hepatic stellate and Kupffer cells may initiate the inflammatory response to induce pyroptosis and liver fibrosis during schistosoma infection (51, 52). The present study demonstrates that stimulation of PSCs can increase the expression of genes associated with pyroptosis, including *NLRP3*, *GSDMB*, *GSDME*, *CASP3* and *CASP7*, in canine EICs. These results suggest that PSCs trigger pyroptosis in canine EICs during the early stages of infection, subsequently inducing an inflammatory response at the infection site. Most pyroptosis induced by parasites is mediated by *GSDMD* (53–55). Nonetheless, our findings suggest that both *GSDMB* and *GSDME* may play a role in pyroptosis of canine EICs induced by PSCs infection. However, further experimental validation is required to elucidate these mechanisms.

*NLRP14*, whose expression was upregulated in the present study, is a notable member of the NLR family. It has a gonad-specific expression pattern, primarily in the testis and secondarily in the ovary (56). Maternal *NLRP14* may play a role in the inhibition of the nucleic acid-sensing pathway and potentially aid fertilization in humans (56). However, the roles of *NLRP14* in the host defense against *E. granulosus* infection are not yet understood. Moreover, the involvement of *NLRP14* in host resistance to other parasitic infections is considerably understudied. The third upregulated NLR, *NLRC3*, exhibited substantially elevated expression levels in CD4+ T cells following PAMP stimulation, indicating its potential functional significance in the host adaptive immune response (57). *NLRC3* expression was upregulated in sheep peripheral blood mononuclear cells following stimulation with a soluble extract from *Haemonchus contortus* (58). These findings indicate that *NLRC3* may be involved in the immune regulatory mechanisms underlying canine resistance to *E. granulosus* infection.

Of the CLRs with upregulated expression, *CLEC4A* has been identified as an immunosuppressive factor in dendritic cells, which orchestrate the adaptive immune response (59). *CLEC17A* may function as a colonization factor in tumor cells, facilitating invasion into the lymph nodes and exhibiting fucosylated motifs that are commonly linked to the epithelial phenotype (60). However, the roles of these CLRs against *E. granulosus* infection in the host remain unknown. In the present study, most GPCRs with upregulated expression belonged to the adhesion GPCR family. These receptors predominantly interact with cell membranes and extracellular matrix proteins as ligands. However, they can also bind to polysaccharides, fatty acids, and phospholipids/glycolipids such as glycosaminoglycans, phosphatidylserine (PtdSer), docosahexaenoic acid, and lipopolysaccharide (61–63). Therefore, we hypothesized that the increased expression of adhesion GPCRs may facilitate their binding to specific molecules of *E. granulosus*, thereby modulating the host immune response during parasite infection.

Our findings demonstrates that PSCs can upregulate the expression of several PRRs downstream signaling molecules in canine IECs. The expression of *IL-5*, *IL-11*, *IL-13*, *IL-25*, and *IL-27* was considerably upregulated at both 12 h and 24 h after PSC stimulation in canine IECs. *IL-5* is involved in the recruitment and activation of eosinophil granulocytes, which can eliminate parasites by releasing toxic molecules such as alkaline proteins (64). Additionally, *IL-13* can enhance mucus goblet cell secretion, promote contraction of intestinal smooth muscles, and facilitate epithelial cell migration and turnover (65). *IL-5* and *IL-13* can also stimulate host cells to increase the levels of IgE, which attracts mast cells and eosinophilic granulocytes to the intestinal wall (66).

*IL-11*, a cytokine belonging to the *IL-6* family, exerts its effects on target cells by interacting with the membrane-bound *IL-11* receptor (67). *IL-11* plays a substantial role in immunomodulation through its action on macrophages/monocytes, CD4+ T cells, and B cells (68). *IL-25*, also known as *IL-17E*, holds a unique position within the *IL-17* family, as it can directly induce type-2 immunity (69). *IL-27* is a cytokine comprising IL-27p28 and EBI3 sub-units, signaling through a receptor with gp130 and IL-27Rα (70). Although *IL-27* can promote T cell responses, mice lacking IL-27Rα or sub-units have stronger T cell responses in various infection models (71–74).

Of the interleukins with upregulated expression, *IL-5*, *IL-13*, and *IL-25* induce type-2 immunity. Additionally, we identified several other upregulated regulators of type-2 immunity, including *ATF6*, *IRF8*, *BCL3*, *NFATC1*, *NFATC2*, and *NFATC3*. *ATF6* is activated by *STAT6* and *STAT3* in Th2 and Th17 cells, respectively. This leads to the increased expression of UPR genes and enhanced differentiation and cytokine secretion in these cell types (75). *BCL3*, an atypical member of the NF-κB family, disrupts the binding of p65-p50 (classical NF-κB) and suppresses the expression of pro-inflammatory cytokines induced by Toll-like receptors. Activation of *BCL3* during *S. mansoni* infection downregulates *IL-12* production while promoting the expression of chemokines that attract Th2 cells, thereby altering T-cell differentiation toward Th2 polarization (76). The NFAT family of transcription factors, including *NFATc1*, *NFATc2*, and *NFATc3*, which are regulated by calcium, is found in both T helper cell subsets. In addition, it works in conjunction with other proteins to markedly contribute to the inducible transcription of numerous genes related to the immune response to antigens (77–79). These findings indicate that the presence of *E. granulosus* in dog intestines may trigger a Th2 immune response.

In summary, this study elucidates the global gene expression patterns in the early stages of *E. granulosus* infection in dogs. RNA-seq and several bioinformatic analyses were used to identify the roles of PRRs in the host innate immune response to the presence of *E. granulosus*. Our preliminary findings elucidate the mechanisms underlying the host innate immune regulation of *E. granulosus* infection in canines. Specifically, *E. granulosus* PSCs upregulated the expression of several PRRs genes, including *TLR1*, *TLR6*, *TLR7*, *TLR10*, *CLEC4A*, *CLEC17A*, *NLRP3*, *NLRC3*, and *NLRP14*. *E. granulosus* PSCs also upregulated the expression of Th2-related transcription factors (*ATF6*, *IRF8*, *BCL3*, *NFATC1*, *NFATC2*, and *NFATC3*). However, further investigation is required to evaluate the potential roles of these PRR ligands and elucidate the specific PAMPs of *E. granulosus* PSCs that are identified by the host immune system.

## Data Availability

The datasets presented in this study can be found in online repositories. The names of the repository/repositories and accession number(s) can be found in the article/Supplementary material.

## Footnotes

^1^https://ftp.ncbi.nlm.nih.gov/genomes/all/GCA/011/100/685/GCA_011100685.1_UU_Cfam_GSD_1.0/

^2^http://www.genome.jp/kegg/
